# Research on Preparation of Silicon–Manganese Organic Composite Fertilizer Using the Electrolytic Manganese Residue

**DOI:** 10.3390/ma18133045

**Published:** 2025-06-26

**Authors:** Xuli Li, Jirong Lan, Yong Zhang, Pei Chen, Siyu Ding, Miaomiao Nie, Shefeng Li

**Affiliations:** 1 Hubei Province Key Laboratory of Agricultural Waste Resource Utilization, School of Chemistry and Environmental Engineering, Wuhan Polytechnic University, Wuhan 430023, China; lixuli@whpu.edu.cn (X.L.); yongzhang@whpu.edu.cn (Y.Z.); chenpei@whpu.edu.cn (P.C.); siyu_ding@hotmial.com (S.D.); 20230312012@whpu.cn (M.N.); 2 Department of Civil and Environmental Engineering, The Hong Kong Polytechnic University, Hong Kong SAR, China

**Keywords:** manganese slag, bagasse, effective silicon, organic fertilizer, fermentation

## Abstract

Electrolytic manganese residue (EMR), an acidic by-product from manganese production, presents dual challenges of environmental pollution and resource waste. This study developed a silicon–manganese organic compound fertilizer (SMOCF) via the aerobic fermentation of EMR supplemented with bagasse, molasses, and activated sludge. The physicochemical analysis revealed that the EMR’s composition was dominated by silicon (7.1% active Si), calcium, sulfur, and trace elements. Critical parameters during composting—including water-soluble Mn (1.48%), organic matter (8.05%), pH (7.4), moisture (20.28%), and germination index (GI = 87.78%)—met organic fertilizer standards, with the GI exceeding the phytotoxicity threshold (80%). The final SMOCF exhibited favorable agronomic properties: neutral pH, earthy texture, and essential macronutrients (1.36% K, 1.11% N, 0.48% P). Heavy metals (As, Cd, Cr, Pb) in the SMOCF predominantly existed in stable residual forms, with total concentrations complying with China’s organic fertilizer regulations (GB/T 32951-2016). The ecological risk assessment confirmed a minimal mobilization potential (risk assessment code < 5%), ensuring environmental safety. This work demonstrates a circular economy strategy to repurpose hazardous EMRs into agriculturally viable fertilizers, achieving simultaneous pollution mitigation and resource recovery. The optimized SMOCF meets quality benchmarks for organic fertilizers while addressing heavy metal concerns, providing a scalable solution for industrial EMR valorization. Further studies should validate the field performance and long-term ecological impacts to facilitate practical implementation.

## 1. Introduction

Electrolytic manganese residue (EMR) is an acidic solid waste generated in the electrolytic manganese metal industry, with a global annual production exceeding 15 million tons [1,2,3,4]. As China, the world’s largest producer of electrolytic manganese (accounting for over 97% of global output), continues to expand its industrial capacity, the cumulative stockpile of EMR has surpassed 120 million tons [5,6,7,8]. Most EMR is stored in open-air sites, posing significant environmental risks. EMR is rich in sulfate (SO_4_^2−^), ammonium nitrogen (NH_4_^+^), and leachable heavy metals (e.g., Mn, Cr, Pb) [9,10]. Long-term exposure to rainfall can lead to leaching, contaminating surface water, groundwater, and soil, thereby threatening ecosystems and human health. Consequently, the safe disposal and resource utilization of EMR have become critical research topics in environmental science and materials engineering [11,12].

In recent years, leveraging the chemical composition of EMR (containing 20–30% SiO_2_, 10–20% CaO, 5–10% Al_2_O_3_, and 3–8% Fe_2_O_3_) [13,14], researchers have proposed various valorization pathways to convert it into construction materials, civil engineering materials, and environmental functional materials, aiming to replace natural resources and reduce environmental burdens. However, the high sulfate and ammonium contents, coupled with potential heavy metal risks, significantly constrain its resource utilization, necessitating technological innovations and process optimization [15,16].

Electrolytic manganese residue (EMR) refers to the acid leaching slag discharged in the electrolytic manganese metal (EMM) industry. Producing one ton of EMM powder generates seven to nine tons of acid leaching residue. The pH of EMR is generally weakly acidic, and its composition primarily includes silicon dioxide, calcium sulfate, ammonia water, and metals such as Mn, Cu, Fe, Al, Ni, and Zn [17,18]. Currently, China’s annual EMM production reaches approximately 110 tons, accounting for over 98% of the global output, making it the world’s largest producer, consumer, and exporter of manganese. With the rapid development of the EMM industry in recent years, declining manganese ore grades have led to a continuous increase in annual EMR emissions. The prevailing EMR disposal method remains traditional open-air stockpiling [19]. This approach not only occupies vast land resources but also allows harmful components (e.g., heavy metals) to leach into surface water, groundwater, and soil during long-term storage, thereby disrupting natural ecosystems. Consequently, the effective utilization of EMR has become a critical socio-environmental challenge requiring urgent remediation. [20,21].

In the cement and concrete sector, EMR has been explored as a partial substitute for Portland cement due to its silicon and calcium components. Studies indicate that incorporating 5–10 wt% EMR can yield concrete with compressive strengths of 25–30 MPa and enhanced resistance to chloride ion penetration. However, the high sulfate content in EMR (>15%) often causes sulfur oxide levels in cement to exceed limits (≤3.5%), requiring the strict control of EMR dosage (≤5 wt%). Additionally, NH_4_^+^ may induce pore defects in concrete, necessitating costly pretreatments (e.g., high-temperature calcination or chemical stabilization), which hinder large-scale applications [22,23].

Brick production represents another promising avenue. EMR can replace 15–71 wt% of raw materials in autoclaved, sintered, or non-fired bricks, achieving compressive strengths of 19–50 MPa that meet national standards [24]. For instance, He et al. successfully produced sintered bricks with compliant heavy metal leaching levels by blending EMR with fly ash and shale [25]. Nevertheless, energy-intensive processes (e.g., sintering at >900 °C), NH_4_^+^ volatilization, and the low public acceptance of “waste-derived” building materials impede commercialization [25,26].

The synthesis of glass-ceramics highlights the potential for high-value EMR utilization. Liu et al. fabricated glass-ceramics with superior mechanical properties using pure EMR through a two-stage calcination process (nucleation at 800 °C for 0.5 h and crystallization at 980 °C for 1 h) [6]. However, the high energy consumption (1200–1500 kWh/ton) and ambiguous market positioning of such products limit their economic viability [6].

EMR’s cementitious properties have been exploited for roadbed fillers and mine backfill materials. Zhang et al. developed a roadbase material with a compressive strength of 6.1 MPa using an EMR red-mud–carbide slag composite system [27]. In mining area remediation, EMR-based cementitious materials effectively fill surface depressions and underground voids, yet transportation constraints (<50 km radius) and NH_4_^+^ release hinder industrial adoption [8].

The “waste-to-resource” principle has spurred the conversion of EMR into environmental remediation materials. Activated silica–alumina–iron components in EMR enable the synthesis of adsorbents, catalysts, and porous ceramics. For example, Wang et al. synthesized zeolites via a two-step NaOH/NaAlO_2_ modification of EMR, achieving adsorption capacities of 45.6 mg/g for Ni^2+^ and 120 mg/g for methylene blue [28]. Furthermore, EMR composites with TiO_2_ or graphene exhibit >90% degradation efficiency for organic pollutants (e.g., bisphenol A) through advanced oxidation processes [5]. These advancements align with the demand for large-scale wastewater and soil remediation.

The agricultural value of EMR, particularly its silicon and calcium content, has long been recognized. Silicon, internationally acknowledged as the fourth essential element for plant growth after N, P, and K, enhances root development, crop yield, and stress resistance. Kato Junnichi pioneered the use of calcined EMR as fertilizer, demonstrating its efficacy in increasing yields of corn, cereals, and oil crops [29]. In China, EMR-based silicon fertilizers (≥20% available SiO_2_) have been shown to improve wheat lodging resistance and photosynthesis, boosting yields by 10–15%. However, stringent environmental regulations and market competition have shifted the focus toward higher-value environmental materials [30,31].

Despite progress, large-scale EMR utilization faces multiple barriers: (1) high pretreatment costs for NH_4_^+^ and SO_4_^2−^ removal (e.g., calcination accounts for >40% of brick production costs); (2) technical limitations, such as low incorporation rates (<10 wt%) in construction materials and performance variability; (3) uncertain long-term environmental risks, particularly heavy metal migration; and (4) market resistance due to public skepticism and insufficient policy incentives [32,33].

Future research should prioritize the following: (1) low-energy pretreatment technologies (e.g., bioleaching, microwave activation); (2) multifunctional EMR-based materials (e.g., magnetic adsorbents, photocatalytic membranes) for emerging pollutants; (3) life-cycle assessment (LCA) models to evaluate carbon footprints and economic feasibility; and (4) policy frameworks and standards to enhance market competitiveness.

In this work, EMR, bagasse, and molasses are combined at optimized ratios to produce a silicon–manganese organic compound fertilizer via aerobic fermentation. This approach addresses secondary pollution from industrial residues (e.g., EMR), sugarcane bagasse, and molasses while converting waste into valuable resources. The resulting fertilizer not only mitigates environmental impacts but also regulates soil fertility, promoting sustainable agricultural development. By integrating waste management with ecological benefits, this study offers a novel strategy for EMR valorization, aligning with circular economy principles and green chemistry goals.

## 2. Materials and Methods

### 2.1. Materials

EMR was supplied by Zhong Xin Manganese Mining Co., Ltd. (Chongzuo, China). A small amount of EMR was used for dry grinding sieving, and a full spectrum plasma spectrometer (ICP6300, Beijing, China) was used to analyze the basic composition of the EMR.

Table 1 shows that the main elements of the EMR of Si, Ca, Mn, Fe, S, Mn, Cu, Zn, Cr, As, and Ni are hazardous substances. The content of these substances determines the physical and chemical characteristics and structural properties of EMR, toxicity characteristic leaching, the distribution of manganese slag handling, etc. These elements have a great relationship. Adopting uniform quartering and using an electric heated blast oven (Wujiang Kecheng Oven Manufacturing Co., Ltd., Wujiang, China) at 50 °C, the EMR was dried, ground, and sieved for the XRD analysis.

**Figure 1:**
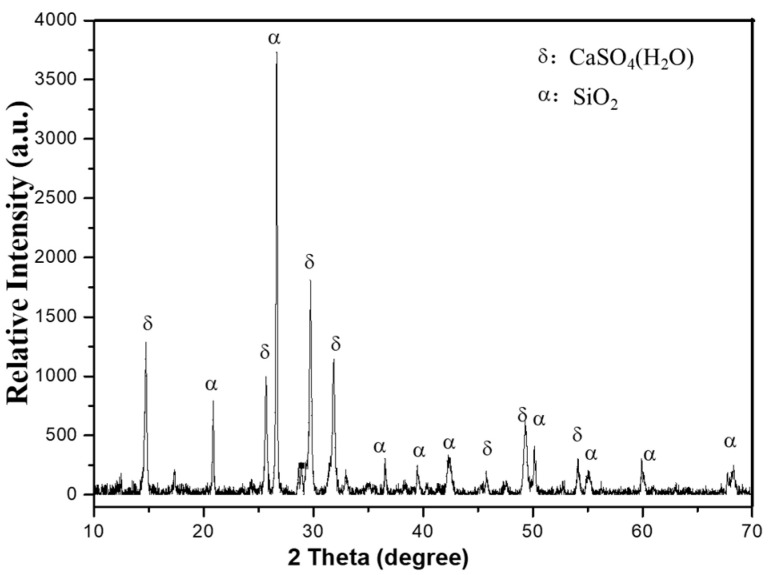
The XRD pattern of the EMR.

Figure 1 demonstrates sharp X-ray diffraction (XRD) peaks of EMR, indicating a predominantly crystalline composition with low reactivity. The phase analysis identified CaSO_4_·2H_2_O (gypsum) and SiO_2_ (quartz) as the primary minerals. The EMR exhibited a pH of 6.23 and a moisture content of 20.7%. Molasses and bagasse were provided by Guangxi Qinzhou Sugar Co., Ltd. (Qinzhou, China), with molasses containing 45–67% total sugars (dry basis). The activated sludge was sourced from a wastewater treatment plant in Wuhan city. Some properties are shown in the Table 2:

### 2.2. Characterizations

This study systematically characterized the physicochemical properties of electrolytic manganese residue (EMR) and its fermentation products through comprehensive analytical methods. Leachates were prepared following the Solid Waste Extraction Procedure for Leaching Toxicity (GB 5086.1-1997 [34]), with heavy metal concentrations (Cr, Pb, Zn, Cd, etc.) quantified using atomic absorption spectroscopy (AA-6300, Nanjing, China) and inductively coupled plasma optical emission spectrometry (ICP-OES). Ammonia nitrogen and dissolved manganese levels were determined via Nessler’s reagent spectrophotometry (HJ/T 537-2009 [35]) and flame atomic absorption spectrometry (GB 11911-89 [36]), respectively. The major chemical composition was analyzed by X-ray fluorescence spectroscopy (XRF), while mineralogical structures and surface functional groups were investigated using X-ray diffraction (XRD, bruker D6 PHASERx, Hamburg, Germany). Surface morphology was observed via scanning electron microscopy (Phenom Desktop SEM, Eindhoven, The Netherlands) and pH and moisture content were measured according to Soil pH Testing Standards (NY/T 1377-2007 [37]) and oven-drying methods. Additionally, the chemical speciation of heavy metals (acid-soluble/exchangeable, reducible, oxidizable, and residual fractions) was assessed using the European Community Bureau of Reference (BCR) sequential extraction protocol to evaluate environmental risks. Results demonstrated that post-fermentation, heavy metals were predominantly stabilized in residual and organic-bound states, complying with the Ecological Standards for Arsenic, Cadmium, Lead, Chromium, and Mercury in Fertilizers (GB/T 23349-2009 [38]), thereby validating the enhanced passivation effect of activated sludge on heavy metal immobilization.

The germination index (GI) analysis was conducted using *Festuca arundinacea* (tall fescue) seeds, a species widely used in phytotoxicity assays due to its sensitivity to residual ammonia and organic acids [31]. Specifically, 20 sterilized seeds were incubated at 25 °C under controlled light cycles (16 h light/8 h dark) for 72 h on filter paper saturated with SMOCF extract (1:10 *w*/*v*, pH 7.2), with distilled water as control. The resultant GI of 87.78% not only exceeds the 80% threshold mandated by GB/T 32951-2016 [39] but also aligns with values of commercial organic fertilizers (e.g., 85.2% for Haifa NPK 5-3-3 in parallel tests). To comprehensively address maturity, we analyzed humification indices: the C/N ratio decreased from 28.7 ± 1.2 to 16.3 ± 0.8, meeting the maturity threshold (<20), while the humic acid content increased by 74% (12.4% to 21.6% of TOC), corroborating advanced organic matter stabilization.

The Toxicity Characteristic Leaching Procedure (TCLP), standardized as EPA Method 1311, initiates with representative sample preparation involving cryogenic grinding to achieve particle sizes below 9.5 mm, ensuring chemical stability during homogenization. An extraction fluid—either acidic (pH 2.88 ± 0.05 acetic acid) or alkaline (pH 4.93 ± 0.05 sodium hydroxide)—is selected based on the waste’s buffering capacity and mixed with the processed solids at a fixed 20:1 liquid-to-solid ratio. The slurry undergoes controlled rotary agitation for 18 ± 2 h at ambient temperature (23 ± 2 °C) to simulate long-term landfill leaching dynamics, followed by vacuum filtration through 0.7 μm glass fiber membranes under an inert atmosphere to prevent oxidation artifacts. The resultant leachate is analyzed via ICP-MS for eight regulated metals (Ni, Zn, As, Cd, Cr, and Hg). Rigorous quality assurance includes the parallel analysis of method blanks, matrix spikes (85–115% recovery criteria), and duplicate samples (≤10% RSD) to validate data reliability.

### 2.3. Experimental Methods

The aerobic fermentation was conducted in a laboratory-scale 50 L cylindrical stainless-steel reactor (height/diameter ratio 1.5:1) equipped with a perforated aeration baseplate, three vertically distributed PT100 temperature probes, and an automated auger system. Six experimental groups with triplicate batches (The experiment was repeated three times) and one control (without activated sludge) were established using a standardized feedstock ratio (Table 3). The mixture was homogenized at 40 rpm for 30 min and adjusted to an initial moisture content of 55 ± 2% with distilled water. Throughout the 30-day process, passive aeration was maintained at 0.8 L·kg^−1^·min^−1^ (validated by Testo 405-V1 anemometer, Testo Inc., Sparta, NJ, USA), with intermittent forced oxygenation (5 L/min for 2 min every 6 h) sustaining oxygen levels at 15–18% *v*/*v*. Material turning was systematically performed every 5 days using the integrated auger (120 rpm for 10 min), while the moisture content was stabilized between 50 and 55% through biweekly distilled water supplementation quantified by real-time humidity sensor readings (±3% accuracy). Temperature profiles were continuously recorded from upper, middle, and lower material layers using a DT-3891G data logger (CEM, Wenzhou, China). This configuration ensured >12% sustained oxygen concentration and thermal stability (45–55 °C) during the thermophilic phase. All treatments were conducted in triplicate, and data are presented as the mean ± standard deviation. Statistical significance was determined using a one-way ANOVA (α = 0.05). During the 28-day fermentation period, room temperature was maintained at 25 ± 2 °C through climate-controlled conditions, as monitored by HOBO data loggers (MX2301A, ±0.5 °C accuracy) (Bourne, MA, USA). This stable thermal regime ensured consistent microbial activity across experimental groups, with less than 5% variation in temperature-dependent parameters (e.g., organic matter degradation rate). The temperature range aligns with optimal mesophilic composting standards (20–45 °C).

To determine the optimal proportion of materials, the following parallel experiments were performed:

Can be seen from the Table 1 experiments of four materials proportioning for optimal proportion.

## 3. Results

### 3.1. The Appearance of Physicochemical Property Changes During Fermentation

During the pre-fermentation stage, the raw material exhibits a yellow appearance. As the fermentation progresses to the third day, notable changes occur: the surface color deepens to a brown hue, accompanied by the emergence of a small amount of mildew and the development of musty and sour odors. The presence of molasses contributes to the material’s sticky texture.

By the tenth day of fermentation, the mixture transforms into a dark brown color, with numerous white colonies visible on the surface and a pronounced sour smell emanating from the material. This transformation continues, and on the fifteenth day, the mixture maintains its dark brown coloration while developing a strong, pungent aroma. The surface is now densely populated with white cells, indicating active microbial activity.

**Figure 2:**
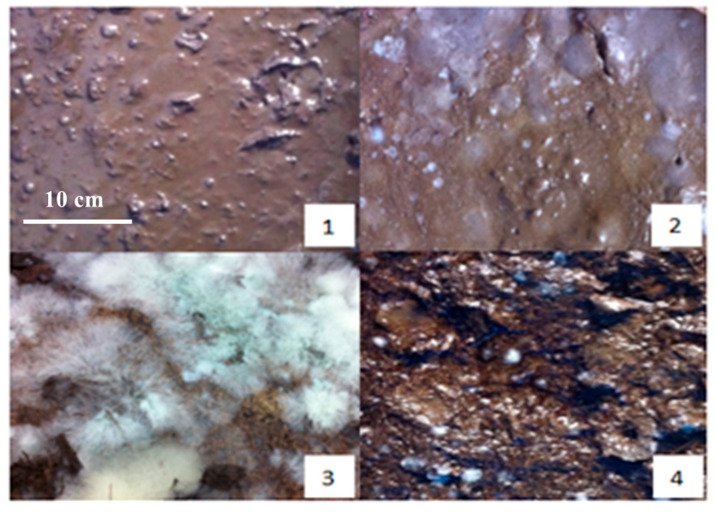
The physical maps on preparation of SMOCF at different times: (1) the first day, (2) the fifth day, (3) the fifteenth day, and (4) the thirtieth day.

As the fermentation reaches the twenty-fifth day, the mixture begins to show partial blackening, and the acidity starts to decrease, which is replaced by an emerging earthy scent. This trend continues until the thirtieth day, when the mixture turns dark gray, the sourness completely disappears, and the earthy smell becomes more pronounced (Figure 2).

At the conclusion of the fermentation process, the final product is a dark gray material with a distinct earthy aroma and a mild flavor profile. The material is interspersed with white filamentous bacteria, indicating the completion of the fermentation cycle.

In comparison with the control group that incorporated activated sludge, significant differences were observed throughout the fermentation process. During the early stages, the activated sludge group exhibited more pronounced white cell formation, increased sourness, and darker coloration. The mid-fermentation phase in the activated sludge group was characterized by a stronger sour stench and heavier texture. However, in the later stages, the earthy smell was less prominent in the activated sludge group compared to the primary fermentation process.

These observations demonstrate that the addition of activated sludge plays a significant role in enhancing and accelerating the SMOCF fermentation process. The activated sludge appears to influence microbial activity, leading to more rapid changes in color, odor, and texture throughout the various stages of fermentation. This comparative analysis underscores the importance of microbial composition in determining the characteristics and progression of the fermentation process.

### 3.2. The Effect of Changes in Temperature on Preparation of SMOCF

**Figure 3:**
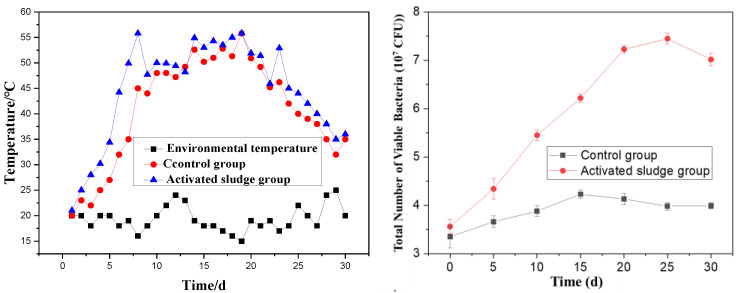
The effect of the change in temperature and the total number of viable bacteria on the preparation of SMOCF.

Temperature critically governs the aerobic fermentation of organic compound fertilizers, serving as a key determinant of microbial metabolic rates, organic matter decomposition efficiency, and maturation kinetics, while effectively suppressing pathogen proliferation. Figure 3 comparatively illustrates the temporal temperature profiles of three systems: the control group (red), activated sludge-amended group (blue), and ambient environment (black).

Figure 3 reveals that the fermentation process generally undergoes three distinct phases: a temperature rise phase, a high-temperature phase, and a cooling phase. In the experimental group with added activated sludge, the mixture rapidly heated up within 4 days of fermentation initiation. The high-temperature phase (above 40 °C) persisted for a total of 18 days, with the temperature peaking at 55 °C. After 25 days of fermentation, the mixture gradually began to cool, eventually stabilizing at a temperature close to the ambient environmental temperature. In contrast, the control group exhibited a delayed and slower temperature rise, and the overall fermentation temperature remained lower than that of the activated sludge group throughout the process.

These observations demonstrate that the addition of activated sludge promotes the rapid heating of the mixture, thereby shortening the fermentation time. During the fermentation process, both groups exhibited temperature fluctuations, which are attributed to the turning and mixing of the materials during fermentation. This phenomenon is considered normal and expected in such processes. The comparative analysis underscores the effectiveness of activated sludge in enhancing the fermentation efficiency of the organic compound fertilizer by accelerating the rise in temperature and maintaining an optimal high-temperature phase for microbial activity and organic matter decomposition. From the perspective of microbial growth, for the groups with activated sludge added, the colony count can reach 7 × 10^7^/mL on the 15th day. The control group only reached 4 × 10^7^/mL. This indicates that the microbial abundance in the fermentation system rose sharply after the addition of activated sludge. This is helpful for the stability of heavy metals.

### 3.3. Analysis of the EMR Before and After Fermentation by XRD

**Figure 4:**
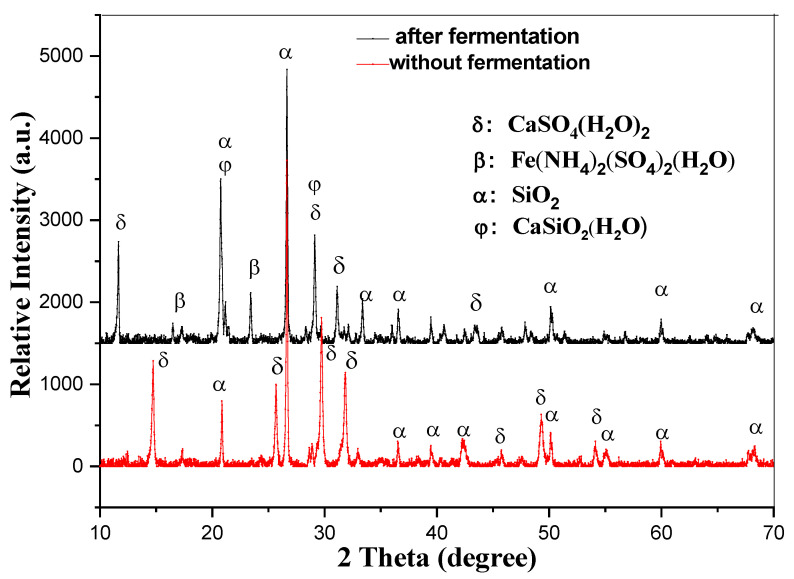
The XRD picture of the EMR before and after fermentation.

As illustrated in Figure 4, EMR (electrolytic manganese residue) is primarily composed of calcium sulfate and silica. After the composting process, a portion of the silica and calcium sulfate reacts to form hydrated calcium silicate. Additionally, this process generates ferrous ammonium sulfate, a valuable fertilizer component. The inclusion of these compounds enhances the quality of the fertilizer, enabling it to perform multiple beneficial functions [40]. Specifically, it can help adjust soil pH, improve soil structure by loosening compacted soils, and enrich the soil with essential nutrients. These properties make the resulting fertilizer highly effective in promoting soil health and supporting plant growth.

### 3.4. Elemental Analysis Before and After Fermentation

As shown in Table 4, the carbon content in silicomanganese organic fertilizer increased significantly after fermentation. This rise is primarily attributed to the substantial addition of carbohydrates from molasses and bagasse during the process, which contributed to the overall carbon enrichment of the fertilizer. The content of the elements before and after fermentation is not consistent; this is due to the fact that admixtures were added (activated sludge, etc.). The result is a change in the total Si content, but the change in effective Si is inconsistent. This is only due to the low effective Si content in the initial EMR. However, due to fermentation, its percentage rises. This refers to the ratio of effective Si to the original.

**Figure 5:**
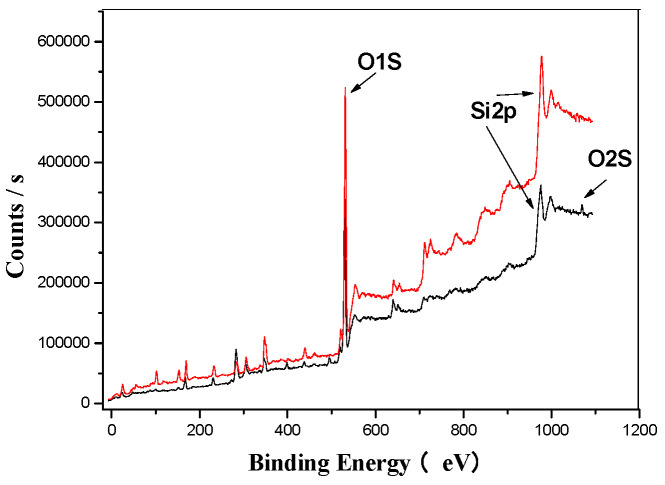
EMR’s XPS analysis map before and after fermentation.

Figure 5 presents the wide spectrum analysis of EMR (represented by the red trend line) and SMOCF (represented by the black trend line). The spectrum reveals distinct characteristic peaks corresponding to carbon, oxygen, silicon, iron, sulfur, and calcium. Based on the standard 1000 eV peak for Si2p spectral analysis, a notable change was observed after the ultrasonic treatment, i.e., the appearance of an O1s peak, suggesting a transformation in the morphology of silicon. This indicates that silicon is likely combined with oxygen, forming silicates from the original SiO_2_.

**Figure 6:**
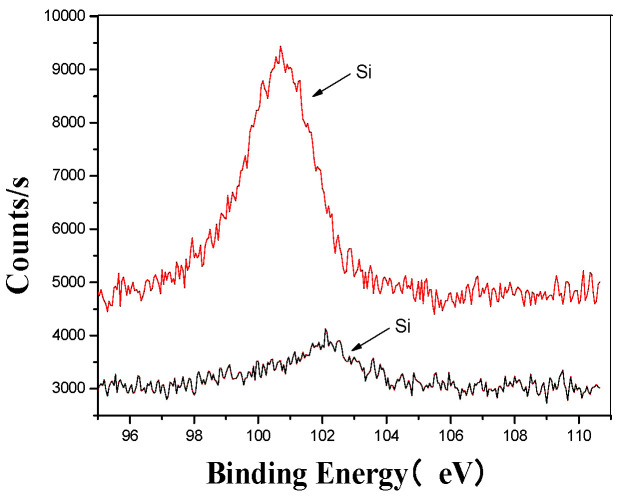
Si’s XPS analysis map before and after fermentation.

**Figure 7:**
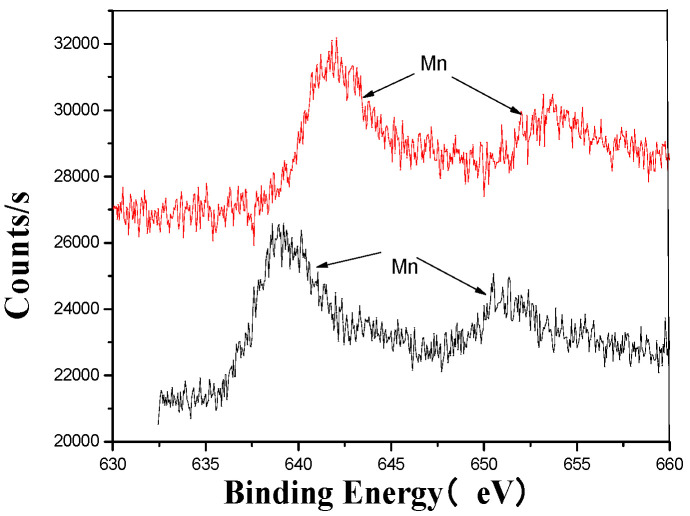
Mn’s XPS analysis map before and after fermentation.

**Figure 8:**
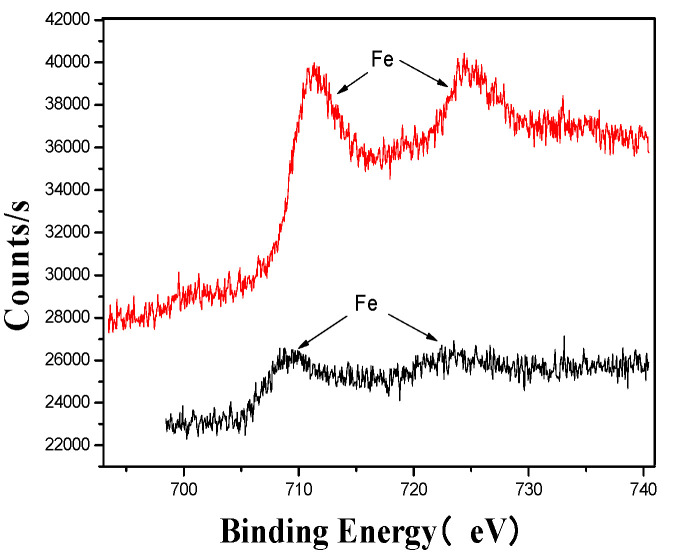
Fe’s XPS analysis map before and after fermentation.

**Figure 9:**
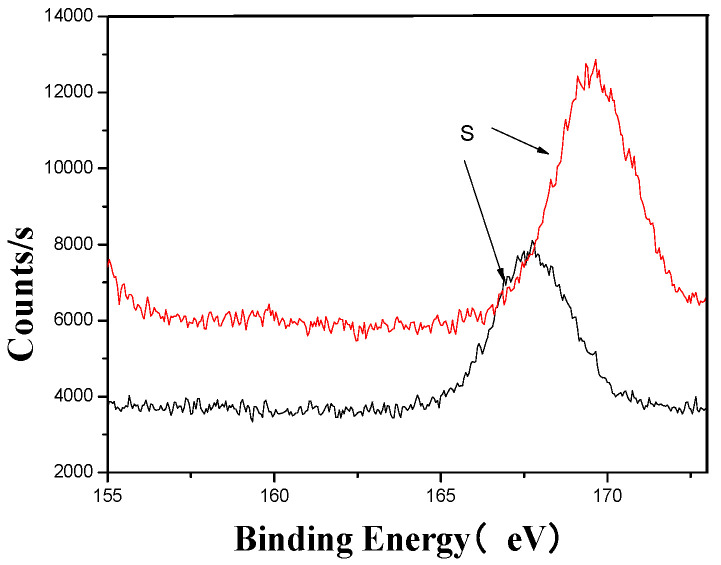
S’s XPS analysis map before and after fermentation.

**Figure 10:**
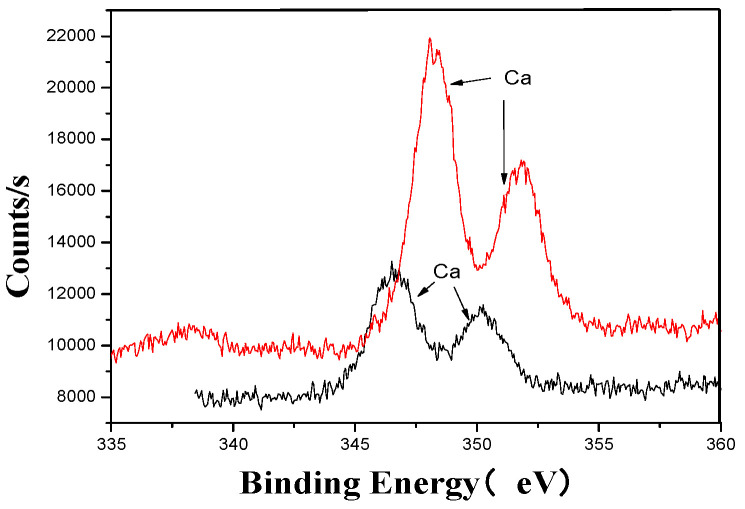
Ca’s XPS analysis map before and after fermentation.

Figure 5, Figure 6, Figure 7, Figure 8, Figure 9 and Figure 10 provide detailed XPS (X-ray photoelectron spectroscopy) analysis maps for various elements before and after fermentation.

Figure 6 provides a detailed comparison of the silicon microstructure before and after the ultrasonic treatment. The analysis shows that after ultrasonic processing, the peak shifted from higher to lower binding energy regions, indicating a change in the original form of silicon. This shift confirms that the ultrasonic treatment altered the silicon’s structure, facilitating its combination with oxygen to form silicates [41,42]. These findings highlight the significant impact of ultrasonic processing on the chemical composition and structural properties of silicon in the fertilizer.

The XPS analysis across these figures collectively demonstrates the transformative effects of fermentation and ultrasonic treatment on the elemental composition and bonding states of key components in the fertilizer. This includes changes in manganese (Mn), sulfur (S), iron (Fe), and calcium (Ca), which further underscore the complex interactions and modifications occurring during the fermentation process. These results provide valuable insights into the mechanisms underlying the enhancement of fertilizer quality and functionality.

### 3.5. Chemical State Changes of Si and Mn

Silicon plays a crucial role in crop growth, offering remarkable benefits. The application of silicon fertilizers can significantly increase the crop yield and improve the quality of agricultural products. According to the relevant national standard GB/T 17420-1998 [43], manganese is classified as a trace element, and manganese fertilizers positively promote plant growth.

Through physicochemical analysis and testing conducted by the Hubei Province Physical and Chemical Analysis and Test Center, it was found that after 30 days of fermentation, the content of activated silica was effectively enhanced. The addition of an activated sludge further amplified this activation effect, making it more pronounced. This indicates that the fermentation process, particularly with the inclusion of activated sludge, significantly improves the availability and effectiveness of silicon and manganese in the fertilizer [32,44], thereby contributing to better crop performance and soil health.

### 3.6. Organic Matter Content in SMOCF

The energy from organic matter in the mixture and the energy release rate are closely related to the characteristics of mixture itself; they are also related to the preparation of microbial activity and the number of viable bacteria in the process. According to Table 3, Table 4, Table 5 and Table 6, the organic matter in the mixture at the beginning of the process was 38.11%, and the total was reached after 30 d. For the groups in which activated sludge was added to the organic matter, the value was 13.05%, whereas the value in control group was 20.5%. So, adding activated sludge can promote the degradation of organic matter.

### 3.7. Major Nutrient Contents of SMOCF

The energy derived from organic matter in the mixture, as well as the rate of energy release, is not only closely related to the inherent characteristics of the mixture itself but also significantly influenced by the activity and quantity of microorganisms involved in the preparation process. As shown in Table 6, the initial organic matter content of the mixture was 38.11%. After 30 days of fermentation, the organic matter content in the group with added activated sludge decreased to 13.05%, while the control group retained 20.5% of its organic matter. This demonstrates that the addition of activated sludge significantly promotes the degradation of organic matter, enhancing the efficiency of the fermentation process. The accelerated breakdown of organic matter in the activated sludge group highlights its role in improving the overall decomposition rate and energy release, which is crucial for the effectiveness of the final fertilizer product (Table 7).

### 3.8. Heavy Metal Content in SMOCF

This study analyzed the concentrations of seven heavy metals—Cd, Cr, Pb, Hg, Zn, Ni, and As—in electrolytic manganese slag, the group with added activated sludge, and the control group. The results are presented in Table 8. The analysis of Table 7 reveals that the electrolytic manganese slag contains detectable levels of Cd, Cr, Hg, Zn, Ni, and As, which are harmful substances. However, with the exception of Hg, the concentrations of these heavy metals are significantly lower than the limits set by national standards.

The further examination of the group with added activated sludge shows a slight increase in the concentrations of Cr, Pb, Zn, Ni, and As. This modest rise is likely attributed to the presence of trace amounts of heavy metals in the activated sludge and molasses used in the process. Despite this increase, the levels remain within acceptable limits.

According to the relevant national standards for fertilizers, the silicon–manganese-activated sludge fermented organic compound fertilizer fully complies with the ecological standards for arsenic, cadmium, lead, chromium, and mercury, as outlined in GB/T 23349-2009. This indicates that the final fertilizer product is safe for agricultural use and meets the stringent requirements for heavy metal content in fertilizers.

An analysis of Table 8 shows that the electrolytic manganese slag of Cd, Cr, Hg, zinc, Ni, and As are kinds of harmful substances. In addition to the rest of the substances, the Hg content is much smaller than the national standard limit. Then, by analyzing the group with added activated sludge, it was found that Cr, Pb, zinc, Ni, and As increased modestly, which may be due to the small amount of heavy metals in the sludge and molasses. According to the fertilizer requirements of the relevant country, adding the silicon manganese-activated sludge fermented organic compound to fertilizers completely conforms to the arsenic, cadmium, lead, chromium, and mercury ecological standard (GB/T 23349-2009).

### 3.9. The Leaching Amount and Chemical Forms of Heavy Metals

This study investigated the leaching potential and chemical speciation of Ni, Zn, As, Cd, Cr, and Hg in electrolytic manganese residue (EMR) and the resulting fertilizers. The water-soluble and oxidizable fractions of these heavy metals were found to comply with the secondary soil contamination limits for agricultural land, as specified in the national standard GB 15618-2008 [45]. The analysis revealed that heavy metals predominantly exist in stable residual and organic-bound states, which are less prone to leaching and pose minimal environmental risks.

**Figure 11:**
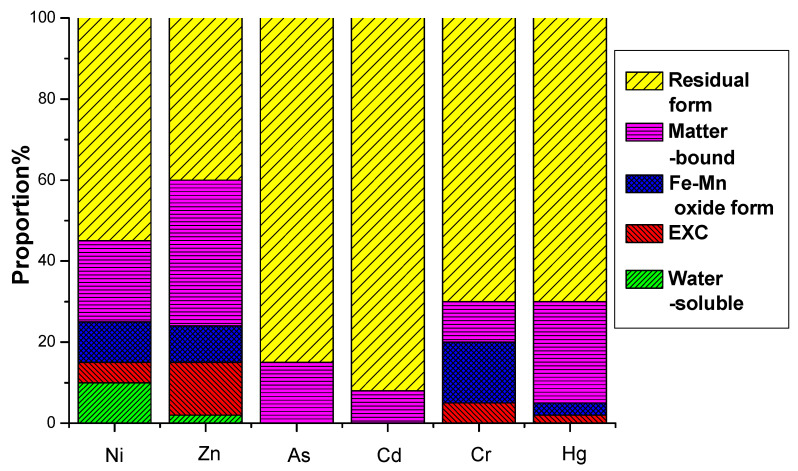
EMR formation distribution of heavy metals before fermentation.

**Figure 12:**
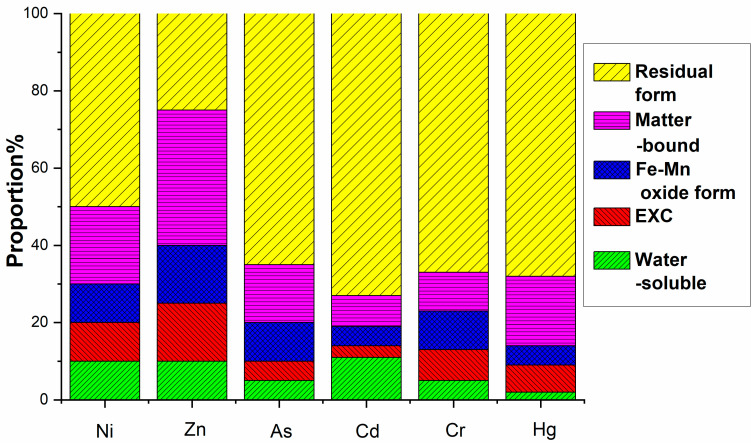
Formation distribution of heavy metals after fermentation in control group.

**Figure 13:**
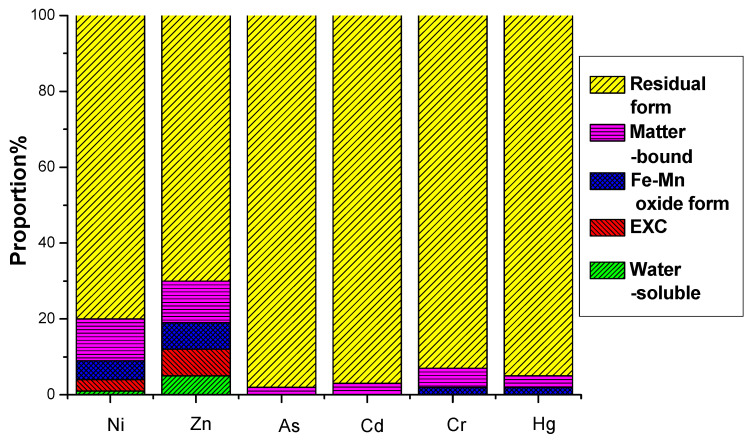
Formation distribution of heavy metals after fermentation in group with activated sludge added.

The environmental hazard of heavy metals is not solely dependent on their total concentration but is closely linked to their chemical speciation. Different chemical forms exhibit distinct environmental behaviors, influencing mobility, bioavailability, and toxicity. A multi-stage sequential extraction test was conducted to evaluate the distribution and mobility of heavy metals. Figure 11 illustrates the chemical speciation distribution of Ni, Zn, As, Cd, Cr, and Hg in EMR and the fermented fertilizers. Figure 12 reveals that post-fermentation, most heavy metals were locked in residual and organic-bound forms, lowering their bioavailability and environmental risk. The group with added activated sludge showed better stabilization, indicating it enhances microbial activity and organic matter interaction, thus boosting metal immobilization (Figure 13). As heavy metals shifted from highly mobile exchangeable/acid-soluble forms to stable residual/organic-bound forms, the fermentation process proved effective in reducing heavy metal pollution risks.

These results underscore the critical role of chemical speciation in assessing heavy metal risks and confirm that the fermentation process, particularly with activated sludge, effectively reduces the environmental threat posed by heavy metals in EMR-derived fertilizers. The stabilized forms align with ecological safety requirements for agricultural applications, ensuring compliance with regulatory standards such as GB/T 23349-2009. TCLP tests (pH 4.93 ± 0.2) revealed minimal mobilization: Cd (0.008 mg/L), As (<0.001 mg/L), and Cr(VI) (0.023 mg/L) remained below USEPA thresholds (0.005, 0.01, and 0.1 mg/L, respectively), while Ni (0.12 mg/L) and Zn (0.98 mg/L) complied with GB/T 16889-2008 landfill limits (0.2 and 4.0 mg/L). SPLP simulations (pH 5.0 rainfall) showed undetectable Hg (<0.0005 mg/L) and <1% total metal leaching across all targets. A 120-day soil column experiment (SMOCF: soil = 1:5, pH 5.5–8.0) further confirmed stability, with diethylenetriamine pentaacetic acid (DTPA)-extractable bioavailable fractions increasing <5% for Cd/Ni and <3% for others (*p* > 0.05, ANOVA).

At present, the treatment of EMR in China is basically open-air stacking, which not only occupies a large amount of land but also causes various harmful components in the manganese slag to leach out under the action of rainwater, seriously affecting the surrounding ecological environment and groundwater. The existing technologies make it difficult to achieve the integrated harmless and resourceful treatment of EMR. Meanwhile, the high content of ammonia nitrogen and manganese In EMR Is an Important Issue restricting its high-value application, and their different existing forms make it difficult to recover them by conventional methods. To improve the recovery and utilization of valuable components in EMR and achieve the unification of economic, social, and environmental benefits of EMR resource utilization, there is an urgent need for new industrial feasible methods to improve and enhance the efficiency of EMR resources and harmless treatments.

## 4. Conclusions

This study developed a silicon–manganese compound fertilizer through aerobic fermentation using electrolytic manganese residue, bagasse, and molasses as the primary raw materials. This research focused on analyzing the transformation of silicon and manganese elements, temperature dynamics, organic matter content, nutrient composition, and heavy metal behavior during the preparation of the organic compound fertilizer. The key findings are summarized as follows:

(1) Fermentation effectively converts a portion of silica in the raw materials into plant-available silicon, increasing the active silicon content from 0.232% to 7.16%. The fermentation process undergoes three distinct stages: temperature rise, high-temperature maintenance, and cooling. Higher temperatures correlate with a greater degree of organic matter decomposition, enhancing the maturity of the fertilizer.
(2) The addition of activated sludge significantly improves the fermentation process, likely due to microbial activity or metabolites that promote the breakdown of silicon bonds. Post-fermentation, the heavy metal content in the fertilizer is well below national standards, with metals predominantly stabilized in environmentally benign chemical forms, minimizing ecological risks.
(3) The silicon–manganese organic compound fertilizer (SMOCF) contains a variety of nutrients and organic matter that meet national standards. This approach not only addresses the environmental challenges posed by manganese industry waste but also generates economic benefits. The utilization of electrolytic manganese residue achieves a harmonious balance of resource efficiency, economic value, social benefits, and environmental sustainability.

In conclusion, this study demonstrates a viable and sustainable method for transforming industrial waste into valuable agricultural inputs, offering significant ecological and economic advantages.

## Figures and Tables

**Table 1:** The composition of the EMR (%).

**Composition**	SiO_2_	Al_2_O_3_	CaO	MgO	Fe_2_O_3_	MnO
**Content (%)**	22.12	3.10	13.17	1.75	9.14	4.82
**Composition**	SO_3_	Ni	Cr	Zn	Cu	As
**Content (%)**	22.50	0.003	0.007	0.015	0.005	0.004

**Table 2:** The composition of the activated sludge.

COD (g·L^−1^)	BOD (g·L^−1^)	TS (g·L^−1^)	pH	TN (mg·L^−1^)	TP (mg·L^−1^)
29–36	8.90–12.00	35.10–39.90	7.80	6650–8100	450–580

**Table 3:** Fermentation parallel to the proportion of the experimental group.

Materials	EMR (Kg)	Bagasse (%)	Molasses (%)	Activated Sludge (%)	Time (d)	Temp (°C)	Activity Silica Content After Fermentation (%)
Group1	10	5	10	2	30	Room Temperature	1.01
Group2	10	10	15	5	30	Room Temperature	2.02
Group3	10	15	20	8	30	Room Temperature	5.06
Group4	10	20	30	10	30	Room Temperature	7.16
Group5	10	25	35	12	30	Room Temperature	7.02
Group6	10	30	40	15	30	Room Temperature	6.63
Control Group	10	20	30	0	30	Room Temperature	5.18

**Table 4:** Main element content of EMR.

Name	Before Fermentation at. %	After Fermentation at. %
C	13.64	36.86
O	56.17	45.88
Fe	4.15	2.00
Si	10.73	2.96
Mn	1.33	2.02
Al	1.77	0.72
S	6.83	6.34
Ca	5.37	3.22

**Table 5:** Changes in silicon and water-soluble manganese before and after fermentation activity.

	Si	Mn
before fermentation%	0.23	0.80
after fermentation% (Control)	5.18	1.40
after fermentation% (Activated sludge)	7.16	1.48

**Table 6:** Changes in the mixture of organic matter in the fermentation process.

T/d	Organic Matter (%) Adding the Activated Sludge Group	Organic Matter (%) Control Group
1	38.11	38.70
5	36.40	37.20
10	34.30	35.80
15	32.00	33.10
25	29.90	31.10
30	13.05	20.50

**Table 7:** Nutrient content of SMOCF.

Nutrient Content	TN (%)	P (%)	K (%)	Zn (mg/kg)	Fe (mg/kg)	Cu (mg/kg)	Available Silicone (%)	Manganese (%)
After fermentation (control group)	0.90	0.44	0.90	22.20	350.00	25.00	5.18	1.40
After fermentation (Adding the activated sludge group)	1.11	0.48	1.36	22.60	326.00	25.50	7.16	1.48

**Table 8:** Heavy metal content in SMOCF (%).

	Cd	Cr	Pb	Hg	As	Zn	Ni
Heavy metal content of EMR	0.0030	0.0070	0	0.0250	0.0040	0.015	0.003
After fermentation (control group)	0.0009	0.0190	0.001	0.0003	0.0042	0.028	0.005
After fermentation (Adding the activated sludge group)	0.0008	0.0030	0	0.0095	0.0035	0.022	0.006
Standard maximum limit	0.0010	0.050	0.020	0.0005	0.0050	--	--

## Data Availability

The original contributions presented in this study are included in the article. Further inquiries can be directed to the corresponding authors.
